# Supplementary figures and images for: Guijing2501 (Citrus unshiu) Has Stronger Cold Tolerance Due to Higher Photoprotective Capacity as Revealed by Comparative Transcriptomic and Physiological Analysis and Overexpression of Early Light-Induced Protein
Source: Int J Mol Sci. 2023 Nov 3;24(21):15956. doi: 10.3390/ijms242115956 (PMC10647585; doi:10.3390/ijms242115956)

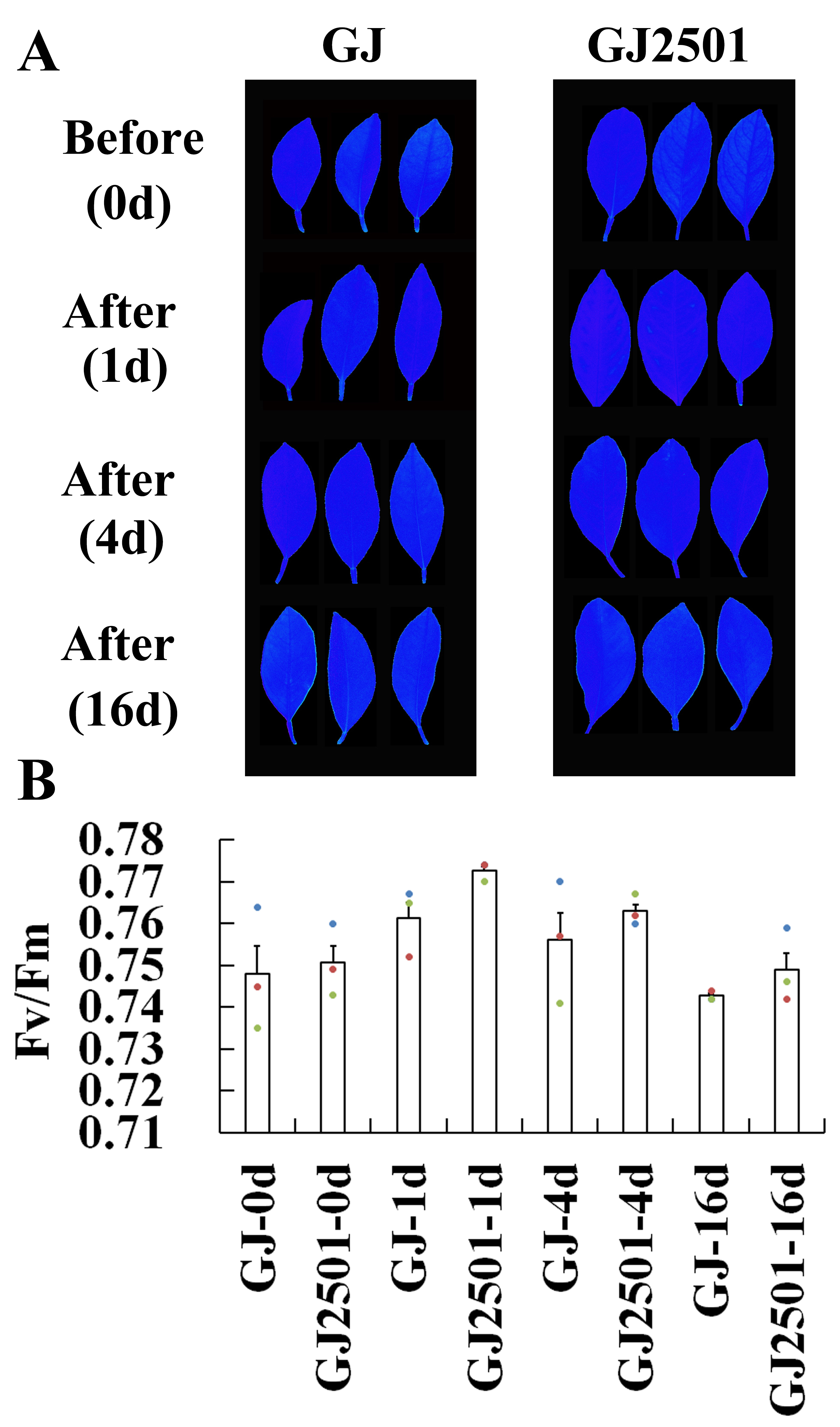

Supplement: Supplementary file 1 [file ijms-24-15956-s001.zip › Supplementary Materials_Proofreading version/Figure S1.jpg]
